# Waist circumference cut-off values for the prediction of cardiovascular risk factors clustering in Chinese school-aged children: a cross-sectional study

**DOI:** 10.1186/1471-2458-10-82

**Published:** 2010-02-19

**Authors:** Ailing Liu, Andrew P Hills, Xiaoqi Hu, Yanping Li, Lin Du, Ying Xu, Nuala M Byrne, Guansheng Ma

**Affiliations:** 1 National Institute for Nutrition and Food Safety, China Center for Disease Control and Prevention, Beijing, China; 2 Institute of Health and Biomedical Innovation, Queensland University of Technology, Queensland, Australia; 3 Guangzhou Center for Disease Control and Prevention, Guangzhou, China; 4 Liaoyang Center for Disease Control and Prevention, Liaoyang, China

## Abstract

**Background:**

Waist circumference has been identified as a valuable predictor of cardiovascular risk in children. The development of waist circumference percentiles and cut-offs for various ethnic groups are necessary because of differences in body composition. The purpose of this study was to develop waist circumference percentiles for Chinese children and to explore optimal waist circumference cut-off values for predicting cardiovascular risk factors clustering in this population.

**Methods:**

Height, weight, and waist circumference were measured in 5529 children (2830 boys and 2699 girls) aged 6-12 years randomly selected from southern and northern China. Blood pressure, fasting triglycerides, low-density lipoprotein cholesterol, high-density lipoprotein cholesterol, and glucose were obtained in a subsample (n = 1845). Smoothed percentile curves were produced using the LMS method. Receiver-operating characteristic analysis was used to derive the optimal age- and gender-specific waist circumference thresholds for predicting the clustering of cardiovascular risk factors.

**Results:**

Gender-specific waist circumference percentiles were constructed. The waist circumference thresholds were at the 90th and 84th percentiles for Chinese boys and girls respectively, with sensitivity and specificity ranging from 67% to 83%. The odds ratio of a clustering of cardiovascular risk factors among boys and girls with a higher value than cut-off points was 10.349 (95% confidence interval 4.466 to 23.979) and 8.084 (95% confidence interval 3.147 to 20.767) compared with their counterparts.

**Conclusions:**

Percentile curves for waist circumference of Chinese children are provided. The cut-off point for waist circumference to predict cardiovascular risk factors clustering is at the 90th and 84th percentiles for Chinese boys and girls, respectively.

## Background

The global prevalence of overweight and obesity has increased dramatically in North America, some European countries, and Australia in recent decades [1-4], however evidence suggests that a greater potential problem exists for China, South America and some countries in North Africa [4,5].

Higher than desirable levels of body fat pose an increased risk of ill-health, however the location of excess fat appears to have particular implications [6-8]. For example, a greater concentration of adipose tissue in the abdomen, specifically in the visceral area, is directly related to metabolic and cardiovascular risk in adults [9]. Visceral adiposity is best quantified using sophisticated imaging techniques however such approaches are not feasible at the population level [10].

Recent attention has been paid to the applicability of anthropometric markers to measure abdominal obesity and the waist circumference is consistently identified as a better measure of cardiovascular risk than the body mass index [6,11,12]. Waist circumference is also recognized as a key component of the metabolic syndrome in both children and adults [13,14]. Waist circumference cut-off points associated with increased risk have been developed for adult men and women, however relatively less work has been undertaken in children and adolescents. A further shortcoming of research to date is that reference standards have more commonly been developed on Caucasian populations and may have limited usefulness to people from different ethnic and racial backgrounds [15,16].

An increasing body of research has explored ethnic differences in body composition in both children and adults [10], but considerably more work is needed. For example, the International Diabetes Federation uses the 90th percentile as a cut-off for waist circumference to define the pediatric metabolic syndrome but has recommended the development of ethnic-, age- and gender-specific normal ranges for waist circumference based on healthy values. In short, the percentiles used as cut-offs for waist circumference should be reassessed when more data are available [14].

A number of studies have developed reference waist circumference percentiles for children and adolescents in different countries [17-23]. To date, three studies have reported age- and gender-specific waist circumference cut-offs in Chinese children and adolescents, two in Hong Kong Chinese children and adolescents [24], and the third in children from Xinjiang province [25]. However, there are regional differences in the body composition of Chinese. People living in North China are taller and heavier than those living in the South due to a combination of genetic and environmental factors [26]. The development of waist circumference percentiles and cut-offs for different groups would be particularly valuable.

Therefore, the purpose of the present study was to develop waist circumference percentiles for Chinese children from both the North and South of the country, and secondly, to explore the optimal waist circumference cut-off values for predicting cardiovascular risk factors clustering in this population.

## Methods

### Subjects

Three cities, Liaoyang in the Northeast and Tianjin in the North of China, and Guangzhou in the South, were involved in this study. Two schools were randomly selected from each city. Height, weight, and waist circumference were measured for all children at each school (aged 6-12 years) and 40% of the participants at each school were selected for the collection of blood samples.

Written consent was obtained from both children and their parents and the study protocol approved by the Ethics Review Committee of the National Institute for Nutrition and Food Safety, China Center for Disease Control and Prevention, and the University Human Research Ethics Committee of the Queensland University of Technology, Australia.

### Anthropometric measurements

Height was measured to the nearest 0.1 cm in bare feet. Body weight was measured to the nearest 0.1 kg with a balance-beam scale with participants wearing lightweight clothing. The body mass index (kg/m^2^) was calculated as weight (kg) divided by the square of height (m). Waist circumference was measured to the nearest 0.1 cm at the mid-point between the lower costal border and the top of the iliac crest with the measurement taken at the end of a normal expiration.

### Cardiovascular risk factors measurement

Blood pressure was measured on the study morning using a random-zero sphygmomanometer after the participant rested for 5 min in a seated position. Two resting blood pressure measurements were taken to the nearest 4 mmHg. A venous blood sample was collected from each participant after an overnight fast. Serum glucose concentration, triglycerides, total cholesterol, and high-density lipoprotein cholesterol, and low-density lipoprotein cholesterol was measured.

### Definition of cardiovascular risk factors clustering

Each participant was classified as having cardiovascular risk factors clustering with 3 or more of the following risk factors [16]: (1) systolic blood pressure and/or diastolic blood pressure ≥ 90th percentiles for age, gender and height recommended by the National Heart, Lung, and Blood Institute (U.S.) [27]; (2) triglycerides ≥ 1.7 mmol/L; (3) high-density lipoprotein cholesterol <1.03 mmol/L; (4) low-density lipoprotein cholesterol ≥ 3.4 mmol/L; (5) fasting glucose ≥ 5.6 mmol/L [14,28].

### Statistical analysis

Continuous data were described as means (± standard deviation). The age and gender differences were tested by t-test. Smoothed age- and gender-specific percentiles were constructed using the LMS ChartMaker Pro software package (The Institute of Child Health, London) for the whole population group. Receiver-operating characteristic analysis was used to explore the diagnostic ability of waist circumference to identify the presence or absence of cardiovascular risk factors clustering among children who provided blood samples. The gender-specific value which maximized both sensitivity and specificity was regarded as the optimal threshold for predicting cardiovascular risk factors clustering among boys and girls. Then the age- and sex-specific waist circumference cut-off values were read directly from the corresponding smoothed percentiles constructed from the whole population group by the LMS method. In addition, odds ratio was calculated using logistic regression analysis adjusted for age to explore the risk of having cardiovascular risk factors clustering among boys and girls who were at the optimal threshold of waist circumference and higher compared with their counterparts. The SAS 8.0 software package was used for analyses. All statistical analyses were two-sided and a p value of < 0.05 was considered statistically significant.

## Results

**Figure 1:**
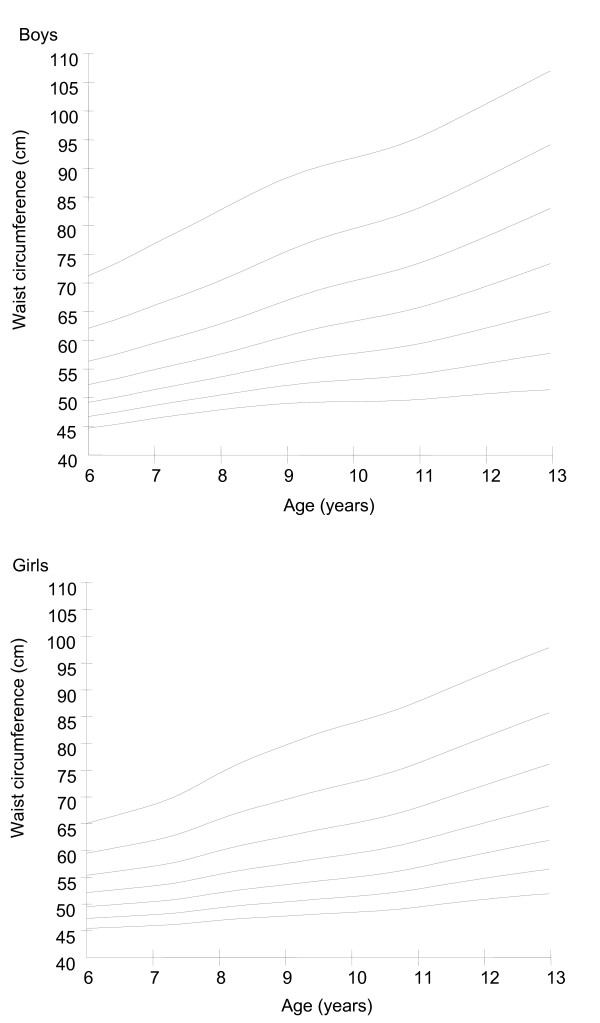
**Smoothed percentile curves for waist circumference in Chinese boys (n = 2830) and girls (n = 2699) aged 6-12 years**.

A total of 5529 children (2830 boys and 2699 girls) aged 6-12 years participated in the study. The descriptive characteristics of the sample by age and gender are shown in Table 1. The age- and gender-specific 3rd, 10th, 25th, 50th, 75th, 90th, and 97th percentiles for waist circumference are shown in Table 2 and smoothed waist circumference percentile curves are presented in Figure 1. In general, waist circumference increased with age in both boys and girls. Boys had a higher waist circumference value than girls at every age and percentile level except for the 3rd, 10th, and 25th percentiles at 6 years.

**Table 1:** Characteristics of the population by age and gender

	n	Height (cm)	Weight (kg)	Body mass index (kg/m^2^)	Waist circumference (cm)
Boys	2830				
Age (years)					
6	152	120.3 ± 5.0	24.1 ± 4.9 *	16.5 ± 2.5*	54.9 ± 7.2
7	469	124.5 ± 5.3**	25.8 ± 5.7**	16.5 ± 2.9**	56.5 ± 7.0**
8	506	129.0 ± 6.3	28.6 ± 8.1**	17.0 ± 3.6**	59.1 ± 8.4**
9	421	134.6 ± 6.8	33.5 ± 10.8 **	18.3 ± 5.4**	62.2 ± 10.1**
10	550	138.8 ± 6.8*	36.4 ± 10.2**	18.6 ± 3.9**	65.5 ± 10.5**
11	499	143.5 ± 7.3*	40.6 ± 11.4 **	19.5 ± 4.4**	67.6 ± 11.0**
12	233	147.0 ± 7.2*	42.7 ± 12.5	19.5 ± 4.4**	68.0 ± 11.8**
Girls	2699				
Age (years)					
6	165	119.6 ± 5.0	22.9 ± 4.2	15.9 ± 2.1	53.8 ± 5.5
7	470	123.1 ± 5.7	24.0 ± 4.4	15.8 ± 2.1	54.0 ± 5.4
8	522	128.7 ± 5.9	27.0 ± 5.6	16.2 ± 2.7	56.6 ± 6.6
9	416	133.5 ± 7.1	30.6 ± 7.5	17.0 ± 3.0	59.3 ± 7.6
10	546	138.0 ± 7.1	33.9 ± 9.1	17.6 ± 3.6	61.3 ± 8.9
11	440	144.3 ± 7.3	38.0 ± 9.1	18.1 ± 3.3	62.8 ± 9.0
12	140	148.5 ± 7.3	41.6 ± 10.1	18.7 ± 3.8	64.3 ± 9.0

*P < 0.05,**P < 0.001 for the difference between boys and girls.

**Table 2:** Waist circumference percentiles (cm) by age and gender

Age (years)	n	3rd	10th	25th	50^th^	75th	90th	97th
Boys	2830							
6	152	44.3	46.5	49.3	52.6	57.0	62.7	71.2
7	469	46.4	48.7	51.5	55.0	59.6	66.1	76.6
8	506	48.2	50.6	53.6	57.4	62.5	70.0	83.1
9	421	49.5	52.4	55.9	60.5	66.6	75.5	90.3
10	550	49.9	53.5	57.8	63.2	70.3	79.9	94.2
11	499	50.3	54.4	59.4	65.4	73.1	83.1	96.8
12	233	51.7	56.6	62.4	69.3	77.7	88.0	101.0
Girls	2699							
6	165	45.3	47.3	49.6	52.3	55.6	59.9	65.6
7	470	45.9	48.0	50.4	53.4	57.1	61.9	68.6
8	522	47.1	49.4	52.2	55.6	60.0	65.9	74.4
9	416	47.9	50.5	53.6	57.5	62.4	69.2	79.3
10	546	48.5	51.4	55.0	59.3	64.9	72.4	83.4
11	440	49.3	52.7	56.8	61.9	68.2	76.6	88.2
12	140	50.4	54.5	59.4	65.4	72.9	82.6	95.5

**Figure 2:**
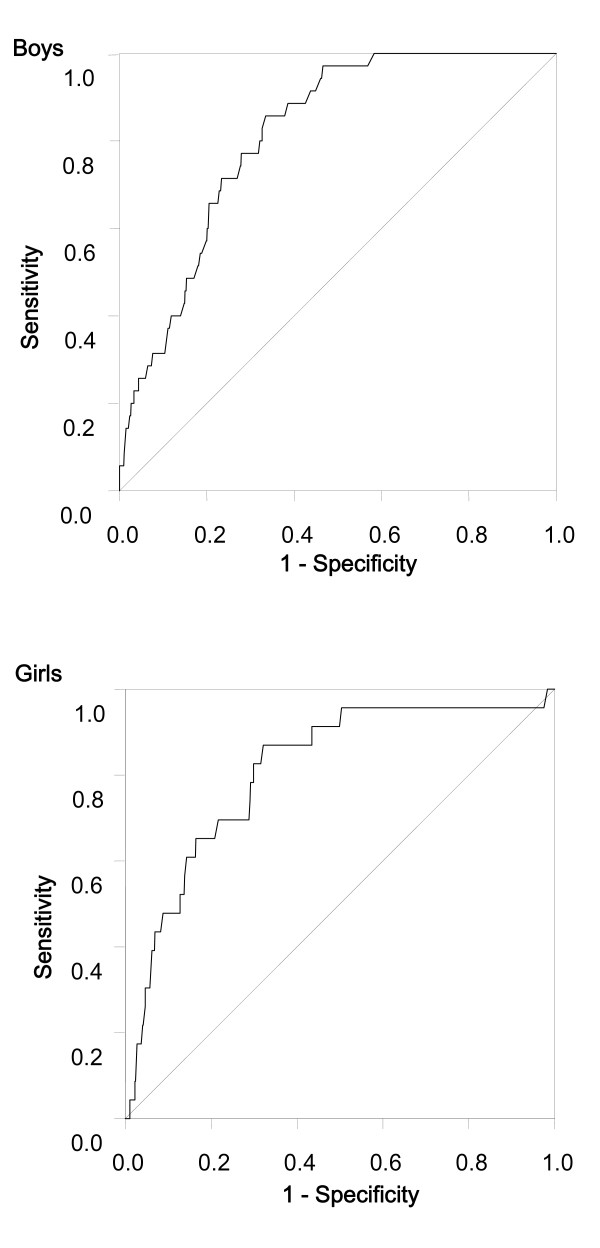
**Receiver-operating characteristic curves for waist circumference with the clustering of cardiovascular risk factors (3 or more of high triglycerides, low high-density lipoprotein cholesterol, high low-density lipoprotein cholesterol, elevated blood pressure, and hyperglycemia) in Chinese boys (n = 982) and girls (n = 863) aged 6-12 years**.

Receiver-operating charactistic curves for waist circumference with high cardiovascular risk (3 or more of 5 cardiovascular risk factors) in boys and girls is shown in Figure 2. Table 3 summarizes the optimal threshold of waist circumference for boys and girls. The 90th and 84th percentiles for waist circumference represented the cut-offs for boys and girls, respectively. The odds ratio of a clustering of cardiovascular risk factors among boys at the 90th percentile of waist circumference and higher, and 84th and higher percentiles of waist circumference in girls was 10.349 (95% confidence interval 4.466 to 23.979) and 8.084 (95% confidence interval 3.147 to 20.767) compared with their counterparts.

**Table 3:** Optimal waist circumference thresholds for cardiovascular risk factors clustering in boys (n = 982) and girls (n = 863)

	Area under the curve (95% confidence interval)	Sensitivity (%)	Specificity (%)	Threshold (percentiles)	Odds ratio (95% confidence interval)
Boys	0.814 (0.761-0.866)	83.3	73.7	90	10.349 (4.466-23.979)
Girls	0.810 (0.720-0.899)	75.0	67.2	84	8.084 (3.147-20.767)

Table 4 shows the age-specific waist circumference cut-off points according to gender. These cut-off points are those above which there is an increased likelihood of being at high risk of cardiovascular risk factors clustering.

**Table 4:** Optimal age- and gender-specific waist circumference cut-off values for Chinese children

Age (years)	Girls	Boys
6	57.2	62.7
7	59.3	66.1
8	62.6	70.0
9	65.7	75.5
10	68.5	79.9
11	71.8	83.1
12	76.3	88.0

## Discussion

This study provides the age- and gender-specific waist circumference reference percentiles for Chinese children aged 6-12 years living in the North and South of the country. Consistent with findings in previous studies [17-21], waist circumference increases with age and boys have a higher value than girls at each age. The age- and gender-related variation of waist circumference also shows similarity to other body dimensions.

**Figure 3:**
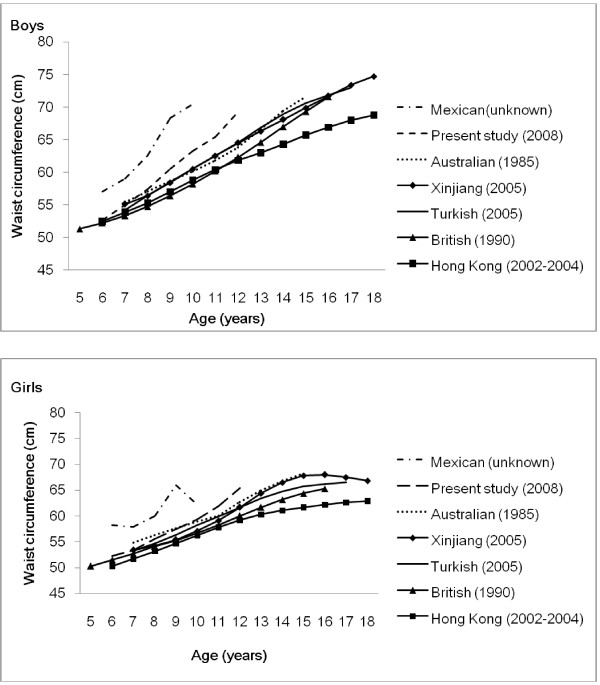
**The 50th percentiles for waist circumference in seven studies for boys and girls**. Data in the present study were collected among children aged 6-12 years in 2008; Data from Australian children aged 7-15 years were collected in 1985 [18]; Data of children aged 7-18 years from Xinjiang province, China were collected in 2005 [25]; Data from Turkish children aged 7-17 years were collected in 2005 [17]; Data from British children aged 5-16 years were collected in 1990 [21]; Data from Hong Kong Chinese children aged 6-18 years was collected in 2002-2004 [24]; Data from Mexican children aged 6-10 years (collection date unknown) [23].

It is interesting to compare our data with those previously reported in British [21], Turkish [17], Australian [18], Mexican [23], and Chinese children in Hong Kong [24] and the Xinjiang Province [25] (see Figure 3). Boys in the present study had higher waist circumference values than Australian, Turkish, and British boys, and lower values than Mexican boys aged 6-12 years, however were similar to British boys aged 6-7 years. Girls in the present study had higher waist circumference values than British, Australian and Turkish girls, and lower values than Mexican girls in all age groups except lower values than Australian girls at 7-9 years. Compared with Caucasians, Asians are generally smaller [29], however waist circumference results from the present study contradict this expectation. A number of factors may contribute to this inconsistency. Firstly, the site of waist circumference measurement has varied between studies and previous research has reported significant differences in waist circumference at different sites [30]. International agreement regarding waist circumference measurement site is required if meaningful comparisons regarding central obesity are to be made between children from different countries and regions. Secondly, considerable changes in body size and shape, including waist circumference, have occurred in recent decades in both children and adults [26,31,32] and this may be a consideration when comparing data collected in different periods. For example, Utter et al. indicated that the mean waist circumference in New Zealand adolescents increased from 76.2 cm in 1997/1998 to 89.4 cm in 2005, with increases in waist circumference measurements at all points in the distribution [31]. Data from Britain and Australia were collected in 1985 and 1990, respectively, a gap of approximately twenty years from the present study. Compared with other studies of Chinese children, both boys and girls in the present study have higher waist circumference values in all age groups however had similar values to 7-year-old children from Xinjiang Province. It is widely accepted that body composition is influenced by the environment in addition to age, gender and ethnicity [10]. Compared with the population in Northern China, both children and adults in Southern China have a smaller body size [26]. This study also demonstrated differences in body size among children in Guangzhou (Southern China), and Liaoning and Tianjin (Northern China) (data not shown). Accordingly, further study is required in more districts of China to generate more representative waist circumference percentiles.

In a sub-sample, the present study also evaluated the threshold value of waist circumference to predict cardiovascular risk factors clustering using receiver-operating characteristic analysis. The 90th and 84th percentiles were identified as the thresholds for diagnosing a higher clustering of cardiovascular risk factors in Chinese boys and girls, respectively. The threshold of waist circumference for boys is consistent with the International Diabetes Federation recommendation [14] and the findings of Maffeis et al. and Ng et al. [15,16], but higher than the two previous studies in Chinese boys. The threshold of waist circumference for girls is lower than the International Diabetes Federation recommendation and similar to the two studies in Chinese girls [24,25]. The thresholds of waist circumference in the present study for both boys and girls are higher than those for Caucasian children [19].

The threshold of waist circumference in previous studies varies according to the definition of cardiovascular risk factors clustering. The two studies from Hong Kong [24] and the study from Xinjiang province [25], proposed the 85th waist circumference percentile as the appropriate threshold for predicting cardiovascular risk. However, Katzmarzyk et al. [19] proposed the 50-57th percentiles for white and black boys and girls. A number of factors may explain these differences. Firstly, waist circumference is ethnic-independent [33,34], as well as the sensitivity to cardiovascular risk factors [35,36]. Secondly, the definition of cardiovascular risk factors clustering has varied between studies with different groupings of cardiovascular risk factors used. Sung et al. reported the 85th percentile was the optimal threshold for diagnosing the presence of cardiovascular risk factors clustering which was defined as 4 or more of 6 risk factors, including elevated systolic and/or diastolic blood pressure, high triglycerides, low high-density lipoprotein cholesterol, high low-density lipoprotein cholesterol, glucose and insulin. However, if cardiovascular risk factors clustering was defined as 3 or more of the 6 risk factors, the cut-offs were the 74th and 69th percentiles for boys and girls, respectively. Furthermore, the level of each cardiovascular risk factor to define an abnormal level also varies between studies, for example, the 75th percentile [37], outer quintiles [19], and 85th percentiles [24]. In the study by Sung et al. [24], the lower the level to define each individual cardiovascular risk factor, the lower the waist circumference percentile. Risk factors tend to cluster together for individuals among both children and adults. The cluster of risk factors and thresholds with the strongest predictive relationship to cardiovascular disease should be identified for both clinical practice and prevention-oriented research and practice for the whole population, especially when developing universal cut-off points to predict it.

## Conclusions

The present study provides waist circumference percentiles for Chinese children aged 6-12 years living in the North and South of the country. Optimal age- and gender-specific cut-off points for waist circumference to predict cardiovascular risk factors clustering are also proposed. Waist circumference percentiles should be updated in different countries consistent with changes in overall body size due to changes in environmental factors. Most importantly, standardized methodology should be used for the development of international waist circumference cut-off points to diagnose cardiovascular risk factors clustering.

## Competing interests

The authors declare that they have no competing interests.

## Authors' contributions

AL participated in the design of the study, collected data, performed the statistical analysis, and drafted the manuscript. APH helped to draft the manuscript and interpret the results. XH participated in the design of the study, collected the data and helped to interpret the results. YL collected the data and helped to perform the statistical analysis. LD participated in the coordination of the study, collected and entered the data, and helped to interpret the results. YX participated in the coordination of the study, collected and entered the data, and helped to interpret the results. NMB helped to draft the manuscript. GM conceived of the study, participated in its design and coordination, helped to perform the statistical analysis and draft the manuscript. All authors read and approved the final manuscript.

## Pre-publication history

The pre-publication history for this paper can be accessed here:

http://www.biomedcentral.com/1471-2458/10/82/prepub
